# Reviews

**Published:** 1882-09

**Authors:** 


### Reviews.

**The Change of Life in Health and Disease:** a Clinical Treatise on the Diseases of the Ganglionic Nervous System, incidental to Women at the Decline of Life. By Edward John Tilt, M. D,, Past President of the Obstetrical Society of London. Philadelphia: P. Blakiston. Son & Co., 1012 Walnut street. Price 75 cents. 1882.

Whatever issues from the pen of Dr. Tilt, in the specialty which he has done so much to develope, is worthy of the consideration of the profession. The field which this work aims to investigate is fertile in the development of pathological conditions, which prove perplexing in their management to the physician and often fatal in their results. Dr. Tilt throws a bomb into the camp of American Surgeons in protesting against the too frequent use of the knife in the treatment of uterine affections, taking the more conservative position peculiar to the English on questions of this kind. This contribution to the literature ot diseases peculiar to the climacteric is worthy of its author, and is valuable in the clear light it sheds upon the physiological and pathological changes, peculiar to the change and the decline of life.

---

**What to Do in Cases of Poisoning.** By WILLIAM MURRELL, M. D., M. R., C. P., Lecturer on Materia Medica and Therapeutics at the Westminster Hospital, etc. Second edition. Detroit, Mich.: Geo. S. Davis, Medical Publisher. 1882.

The object of this little work is to give plain straightforward directions for the treatment of the commonest cases of poisoning. It is an excellent little book and though so small as to be easily carried in vest pockets, it gives the recognized antidotes and directions for the treatment of every case of poisoning which the physician is likely to meet with in active practice.

---

**An Index of Comparative Therapeutics;** with Tables of differential Diagnosis, a pronouncing Dose List in the Genitive Case, a list of Medicines used in Homœopathic Practice and Memoranda concerning Thermometry, Incompatibility of Medicines, Ethics, Obstetrics, Urinary Examinations, etc., etc. By SAMUEL O. L. POTTER, A. M., M. D., President of the Milwaukee Academy of Medicine. Second edition. Chicago: Gross & Delbridge. 1882.

When the first edition of this work appeared, we commented upon its good points and pointed out that it was at least a valuable index to many great works upon therapeutics. A second edition after a few months shows that there are not a few who appreciate its merits.

---

**A Practical Treatise on Diseases of the Skin.** By LOUIS A. DUHRING, M. D. Third edition. Revised and enlarged. Philadelphia: J. B. Lippincott & Co. 1882.

In a previous number of the JOURNAL the second edition of Dr. Duhring's work was favorably reviewed. That in this brief period the third edition should be called for, is a most flattering expression of appreciation by the profession of the merits and value of this book. The present edition has been greatly improved. The chapter on the anatomy and physiology of the skin has been re-written, recent studies in microscospic anatomy demanding the change; while additions in the way of cases illustrating rare and interesting forms of cutaneous diseases, with new and important observations—personal experiences and therapeutics—make the present edition the most valuable addition to the literature of dermatology which has come to our notice. Dr. Duhring has placed the profession under obligations for his labors in presenting a complete series of plates, illustrating the varieties of skin diseases. These plates, with this work, form the most complete guide to the study of dermatology yet offered by any American author. We recommend the work as the most complete and valuable treatise in the department of dermatology, and a medical library can hardly be considered furnished without it.

---

**Atlas of Human Anatomy**; containing 197 large plates. Arranged according to Drs. Oesterreicher and Erdl from their original designs from nature and many other of the greatest anatomists of modern times. With full and explanatory texts by J. A. JEANCON, M. D. Complete in 49 parts, at 75 cents each. Cincinnati, Ohio: A. E. Wilde & Co., Publishers.

It is with no little pleasure that we announce the completion of this great work. The promises of the publishers have been more than fulfilled. No expense has been spared in the production of the plates, many of which are almost life-size. Considering the immense expense of publishing such a work, the price is exceedingly low and within the reach of every practitioner. To those who can not have the advantage of easy access to the dissecting room it is of the greatest value, not only as an aid in keeping up his knowledge of an tomy but as a book of reference.

The Atlas is accompanied with a volume of nearly 200 pages of explanatory text, so arranged as to make the description of any plate easy of access. The work is deserving of all praise, and we hope the enterprise of the publishers will meet a due reward.

**Atlas of Gynecology and Obstetrics.** Edited by Dr. A. MARTIN, Prof. of Gynecology at the University of Berlin. Containing 475 black and 37 colored illustrations from the original designs of Beigel, Virchow, Hyrtl, Kilian, Naegele, Schroeder, Rokitansky, Spiegelberg and 83 others. Supplemented by numerous illustrations from J. P. Maygrier's Nouvelles Demonstrations D'Accouchements. Cincinnati, Ohio: A. E. Wilde & Co., Publishers.

The engraving and typographical work is excellent. The work is published in 15 numbers quarto. The price is one dollar a number or fifteen dollars for the complete work. For the present we feel that we cannot do better than to give to our readers the following preface:

"In placing before the American profession the 'Atlas of Gynæcology and Obstetrics,' it is our desire to give the Students and Physicians a thoroughly practical and demonstrative work on those two important and kindred branches of medical science. While there is no lack of text-books on these subjects, the ordinary octavo size in which these books are printed makes it impossible that they should contain large plates—indeed, many of the cuts are so small as to be obscure—hence, the 'Atlas of Gynæcology and Obstetrics' comes into the field as a Handbook, adapted to and in many respects supplementary to the text-books and treatises already in use.

"Martin's 'Hand-Atlas der Gynæcologie und Geburtshülfe,' a German work, whose large sale in Europe attests its value, if that were not assured, by the illustrious name of its compiler, and Maygrier's 'Nouvelles Demonstrations D'Accouchements,' an Atlas of Midwifery, very popular in France, constitute with some additional plates this Atlas. We believe this is the only series of plates before English readers fully illustrating diseases of woman and obstetrics. Many of the plates are colored, which adds much to their value, since in anatomy it enables one at a glance to distinguish the various structures, and in pathological conditions it gives the best idea which can be had of morbid appearances, outside of the post-mortem room.

"The text to the gynæcological portion is a translation of that contained in Martin's work; the text to the plates on obstetrics has been written for this Atlas, and while no claim is made to originality, the writer has endeavored by diligent study of the modern authorities to give the main points treated in the plates correctly, and to make the text concise, yet clear. The explanatory text is placed opposite the plates—an advantage which every reader will appreciate.

"A glance at the Atlas will show its completeness. In the division relating to gynæcology, the normal anatomy of the uterus, gross and microscopical; the relations of the uterus to surrounding organs; the diseases affecting the genito-urinary apparatus in the female; flexion and version of the uterus; malformations and tumors of the uterus and vagina, are among the principal subjects treated. In the obstetrical division, the signs of pregnancy, the course of pregnancy, the mechanism of normal labor, impediments to delivery, the various presentations, normal and abnormal, and operative obstetrics, are among the subjects considered. Every teacher of obstetrics will recognize at once the usefulness of the plates in clearing up a study which at best is difficult and complicated to the student.

"The very large sale which the 'Atlas of Gynæcology and Obstetrics' has had in its issue in parts, and the flattering notices of the medical press, make the publishers confident that now, when after much labor and great outlay, the work is complete, it will find its proper place as a help and guide in the study of these branches in which America has always held such a leading place."

